# *Glycyrrhiza glabra* L. Extracts Prevent LPS-Induced Inflammation in RAW264.7 Cells by Targeting Pro-Inflammatory Cytokines, Mediators and the JAK/STAT Signaling Pathway

**DOI:** 10.3390/foods14213746

**Published:** 2025-10-31

**Authors:** Maria Rosaria Perri, Michele Pellegrino, Claudia-Crina Toma, Pierfrancesco Prezioso, Vincenzo Tagliaferri, Mariangela Marrelli, Filomena Conforti, Giancarlo Statti

**Affiliations:** 1 Department of Pharmacy, Health and Nutritional Sciences, University of Calabria, 87036 Rende, Italy; mariarosaria.perri@gmail.com (M.R.P.); michele.pellegrino@unical.it (M.P.); pieroprezioso8@gmail.com (P.P.); vincenzo.tagliaferri@unical.it (V.T.); mariangela.marrelli@unical.it (M.M.); filomena.conforti@unical.it (F.C.); 2 Pharmacognosy Department, Faculty of Pharmacy, Vasile Goldis Western University of Arad, 310045 Arad, Romania; claudiatoma2004@yahoo.com

**Keywords:** *Glychyrriza glabra*, inflammation, TNF-α, IL-6, nitric oxide, HPLC, isoliquiritigenin, glycyrrhizin, 18β-glycyrrhetinic acid

## Abstract

*Glycyrrhiza glabra* L. is a species widely spread all over the world, with a long tradition of use in folk medicine. Here, raw and hydrolyzed extracts obtained from roots collected in different geographical areas belonging to the Mediterranean basin were standardized as regards the amount of three main compounds: glycyrrhizin, the most abundant triterpene saponin of licorice, the 18β-glycyrrhetinic acid and the chalcone isoliquiritigenin. Raw and hydrolyzed extracts, as well as their pure single compounds, were investigated for their potential anti-inflammatory properties. The hydrolyzed extracts significantly reduced the production of pro-inflammatory cytokines such as TNF-α, IL-6, NO mediator in LPS-stimulated RAW 264.7 cells. Moreover, they were able to inhibit JAK2 and STAT3 phosphorylated proteins more than pure single standards tested at the same final concentrations, displaying a strength synergism of action. These findings suggest that *G. glabra* extracts and, more specifically, the hydrolyzed ones could represent interesting sources of potential anti-inflammatory agents able to inhibit the JAK/STAT signaling pathway.

## 1. Introduction

Plants have always represented a rich source of bioactive compounds with potential nutraceutical and pharmacological properties. Currently, the demand for natural origin products is dramatically increasing, and the power of plant-based products is attributable to their bioactive constituents including triterpenoids, saponins, flavonoids, etc. [1,2,3,4,5].

Plants from the Mediterranean basin constitute a library of valuable plants, among which *Glycyrrhiza glabra* L. (licorice), belonging to the Fabaceae (Leguminosae) family, represents one of the most ancient and important species. It is an herbaceous perennial plant growing in European countries, India and China [6]. The licorice root apparatus consists of a taproot composed of 3–5 secondary roots, each about 1.25 cm long; root and rhizome barks are green/brown colored. Flowers are narrow, ranging in color from purple to blue, while fruits are small legumes. *G. glabra* has a long tradition in folk medicine; its recognized pharmacological properties include antitussive, antiulcerogenic, anticancer, antidiabetic, anti-asthmatic, anti-atherogenic, anticoagulant, antispasmodic, anti-microbial, antioxidant, anti-inflammatory and anti-allergic activities [7]. The major problem related to licorice intake is “pseudohyperaldosteronism”, a phenomenon causing increased mineralocorticoid activity because of the 11 beta-hydroxysteroid dehydrogenase (11β-HSD2) inhibitory activity. This effect is due to the presence of glycyrrhetinic acid, the most abundant phytochemical in *G. glabra* [8]. Despite this, activity can be observed even at a very low serum concentration. The Food and Drug Administration (FDA) has declared licorice as “GRAS” (Generally Recognized As Safe) for food use and it is also included in some over-the-counter drugs. Even if an official Admitted Daily Intake (ADI) does not exist, different organizations all over the world have established some guidelines for safe use of licorice [9]. Currently, the European Medicine Agency (EMA) is discussing a draft concerning *Liquiritiae radix* (licorice root), with the aim of creating a monograph including information about its qualitative and quantitative composition, therapeutic indications, posology, contraindications, special warnings, precautions for use and interaction and effects on fertility, pregnancy and lactation [10]. The idea behind this work was to partially mimic what usually happens inside the human body by removing glycyrrhizin from the extracts through a hydrolysis process. Indeed, evidence has shown that, both in humans and in rats, a glycyrrhizin peak was not detected in plasma by high performance liquid chromatography (HPLC) after oral administration because *Streptococcus* and *Eubacterium*, thanks to their β-glucuronidase activity, are able to hydrolyze glycyrrhizin into its aglycone form [11]. Glycyrrhizin, a triterpene saponin, and its corresponding aglycone, the triterpenoid glycyrrhetinic acid, are the most investigated phytochemicals of *G. glabra*, known for their antioxidant, anti-inflammatory, antiviral, antitumor and immunoregulatory properties [12]. More than 400 phytochemicals have been isolated from licorice and most of them are flavonoids. The chalcone isoliquiritigenin (2′,4′, 4-trihydroxycalchone), a phytochemical belonging to the flavonoid family, is a naturally occurring pigment in licorice roots and shallot [13] which exerts a wide range of bioactive activities among which are anti-inflammatory properties [14]. In this frame, the aim of this work was to investigate and compare the potential anti-inflammatory activity of both pure single standards and the whole *G. glabra* extracts.

In fact, both the role of inflammatory process onset and the consequences it drives, have been currently well established: evidence has shown how chronic inflammation plays a key role in cancer development, and epidemiological studies have demonstrated that non-steroidal anti-inflammatory drugs (NSAIDs) can be considered effective chemo-preventive agents [15,16]. In the context of prevention, the inhibition of pro-inflammatory responses promoted by the JAK/STAT signaling pathway modulation is a very promising approach. In fact, the activation of STAT transcriptional factors induces the creation of a pro-tumorigenic microenvironment and promotes cell differentiation and renewal. STAT3 aberrant activation is considered a key player in tumorigenesis initiation and progression processes: it acts as a transcription inducer affecting not only gene expression but also contributing to the formation of the tumor microenvironment [17].

## 2. Materials and Methods

### 2.1. Reagents

RAW 264.7 cells were purchased from ATCC, Glasgow, UK (No. TIB-71). Dulbecco’s Modified Eagle’s Medium (DMEM), fetal bovine serum (FBS), L-glutamine, penicillin/streptomycin, phosphate buffered saline (PBS), bovine serum albumin (BSA), protease inhibitors, trypan blue, lipopolysaccharides (LPS) from *E. coli*, trichloroacetic acid (TCA), sulphorhodamine B (SRB), Tris base, isoliquiritigenin, glycyrrhizin and 18β-glycyrrhetinic acid were obtained from Sigma-Aldrich S.p.a. (Milan, Italy). ELISA kits were from ThermoFisher Scientific (Vienna, Austria). The ECL System was from Bio-Rad (Milan, Italy). Anti-phosphoJak2 (#PA5120138), anti-Jak2 (#PA511267), anti-phosphoSTAT3 (#PA5121259) and anti-STAT3 (#PA5120138) were from ThermoFisher Scientific. Anti-β-actin (AC-15; sc-69879) was from Santa Cruz Biotechnology, Inc., Heidelberg, Germany. Solvents were HPLC-grade and reagent-grade, purchased from VWR International s.r.l. (Milan, Italy).

### 2.2. Plant Material Recovery, Extraction Procedure and Hydrolysis Process

*G. glabra* L. investigated extracts were from the Mediterranean area: Morocco, Southern Italy (Rossano (CS) and Montalto Uffugo (CS)). As detailed in Table 1, while one root sample was collected in October (leg. det. G. Statti), others were commercially and locally available. All samples were properly dried at room temperature and shredded before the extraction process by means of a disintegrator, then passed through a 600 µm sieve [18]; pulverized samples were extracted through ultrasonication (MeOH 80%, 20 mins, 25 °C) according to Zhang and colleagues’ method (2013) [19]. Achieved yields were 7.2%, 6.8%, 6.7% and 6.5% for the MON, ROS1, ROS2 and MAR samples, respectively. An aliquot of each obtained extract was dried, while the others were subjected to the hydrolysis process through the addition of HCl (100 °C, 60 min, water bath). Then, the obtained mixture was extracted in ethyl acetate, and the organic solution was mixed with NaCl. The organic phase, dried with Na_2_SO_4_, was filtered and dried using a rotary evaporator.

### 2.3. HPLC Analyses

The three main phytochemicals characterizing the *G. glabra* species, isoliquiritigenin, glycyrrhizin and 18β-glycyrrhetinic acid, were researched and quantified within both the raw and hydrolyzed extracts. Analyses were carried out in a Kinetex XB-C18 250 × 4.6 mm 5 µm column (Phenomenex Inc., Torrence, CA, USA), with a gradient mobile phase consisting of water 0.1% formic acid (solvent A) and acetonitrile 0.1% formic acid (solvent B), as reported by Zhang and colleagues (2013) [19]. The linear gradient separation applied was as follows: 25–45% B from 0 to 30 min, 45–100% B from 30 to 60 min. The instrument was equipped with an MD2010 Plus PDA detector (Jasco, Hachioji, Tokyo, Japan) working at 255 nm, 370 nm and at maximum absorbance between 210 nm and 410 nm. Data were interpolated with standard calibration curves of isoliquiritigenin, glycyrrhizin and 18β-glycyrrhetinic acid, respectively.

### 2.4. Cell Culture

RAW 264.7 macrophages were cultured in DMEM implemented with FBS, 1% L-glutamine and 1% penicillin/streptomycin and incubated at 37 °C in 5% CO_2_ atmosphere [20]. Cells were tested monthly for mycoplasma negativity (MycoAlert Mycoplasma Detection Kit, Lonza, Gampel-Bratsch, Switzerland).

### 2.5. Cell Viability (SRB) Assay

The sulphorhodamine B (SRB) assay, a widely standardized test evaluating cell protein content, was performed in order to investigate cell viability. To start, 3000 cells/well were seeded into a 96-well/plate and incubated overnight to favor attachment. After 24 h, cells were treated with proper concentrations of samples (in DMSO 0.5%) and, after 30 min, stimulated with LPS from *E. coli* (1 µg/mL). The next day, the cells were fixed with TCA 10% (1 h, 4 °C), treated with an SRB solution and washed with acetic acid 1% (three times). The absorbance was measured at 540 nm using a microplate reader (Stat fax 3200 Awareness Technology Inc., Palm City, FL, USA) [21].

### 2.6. Cytokine Measurements

To start, 3 × 10^5^ RAW 264.7 cells/well were seeded into a 24-well/plate and incubated overnight. After 24 h, cells were treated with samples and stimulated with LPS, as reported above. The next day, media was collected, and the presence of pro-inflammatory Tumor Necrosis Factor-α, Interleukin-6 (TNF-α, IL-6) and anti-inflammatory cytokine Interleukin-10 (IL-10) was assessed by means of ELISA kit according to the manufacturer’s instructions (ThermoFisher Scientific, Bender MedSystems GmbH, Vienna, Austria) [22].

### 2.7. Nitric Oxide (NO) Production Inhibition

In order to evaluate the presence of nitrite, a stable oxidized NO product, 3 × 10^5^ RAW 264.7 cells/well were seeded into a 24-well/plate for 24 h, and then they were treated with samples and stimulated with LPS (1 µg/mL). The next day, the collected medium was mixed with Griess reagent (media: Griess reagent, 1:1). The absorbance was measured at 550 nm with a microplate reader (Stat fax 3200 Awareness Technology Inc., Palm City, FL, USA) [23].

### 2.8. Western Blot Analysis

Treated and LPS-stimulated RAW 264.7 cells were lysed in a RIPA lysis buffer [20 mM Tris-HCl (pH 7.5), 150 mM NaCl, 1 mM Na2 EDTA, 1 mM EGTA, 1% NP-40, 1% sodium deoxycholate, 2.5 mM sodium pyrophosphate] supplemented with phosphatase and a protease inhibitor cocktail. Equal lysed protein amounts were run on SDS-PAGE 11% gel and electroblotted onto a nitrocellulose membrane. Proteins were revealed through specific polyclonal or monoclonal antibodies (Abs) and recognized by IRDye secondary Abs (LI-COR Corporate, Milan, Italy). Densitometry readings/intensity ratio was assessed by using ImageJ software 1.53k (Rasband, W.S. ImageJ, U.S. National Institutes of Health, Bethesda, MD, USA) [24].

### 2.9. Statistical Analyses

Data were reported as mean ± S.E.M. of four independent experiments (for SRB assay) and three independent experiments for all the other experiments, each performed in three technical replicates. Phytochemical data were expressed as mean ± S.D. Normality of data and homogeneity of variances were assessed by D’Agostino–Pearson’s K2 test and Levene’s test, respectively, in order to satisfy ANOVA assumption criteria. Statistical differences among groups and treated groups were investigated by Dunnett’s multiple comparison test via one-way ANOVA. Western blot images were analyzed through ImageJ (Rasband, W.S. ImageJ, U.S. National Institutes of Health, Bethesda, MD, USA).

## 3. Results and Discussion

### 3.1. HPLC Analyses

The content of the secondary metabolites may vary among different licorice populations, as also underlined by Esmaeili and colleagues, who reported the quantitative variability of glycyrrhizic acid, glabridin, liquiritin and liquiritigenin content in 22 *G. glabra* populations from Iran [25]. Moreover, the findings from Eghlima and colleagues pointed out that the location where licorice is grown significantly influences its chemical characteristics, influencing the glycyrrhizic acid and liquiritigenin content [26]. Here, through HPLC analyses, raw and hydrolyzed *G. glabra* extracts were standardized in the content of three main bioactive compounds: 18β-glycyrrhetinic acid, glycyrrhizin and isoliquiritigenin (Table S1). The highest content of 18β-glycyrrhetinic acid and isoliquiritigenin was assessed in the hydrolyzed extracts while, as expected, glycyrrhizin was not detected. The triterpenoid saponin glycyrrhizin, in fact, undergoes the attack of some intestinal bacteria, which transform it in its aglycone form, glycyrrhetinic acid [27]. The highest amount of 18β-glycyrrhetinic acid was detected in MON H hydrolyzed extract (40 ± 0.834 µg/1 mg of extract), consistently with the highest amount of glycyrrhizin detected in the corresponding raw extract (MON 78.04 ± 0.76 µg/1 mg of extract). As reported, isoliquiritigenin was found in all the investigated *G. glabra* samples, both raw and hydrolyzed, with the highest amount in ROS2 H samples (38 ± 0.036 µg/1 mg of extract, *p* < 0.05 Bonferroni post hoc test).

### 3.2. Effects of G. glabra L. Extracts and Standards in RAW264.7 Cell Viability

**Figure 1:**
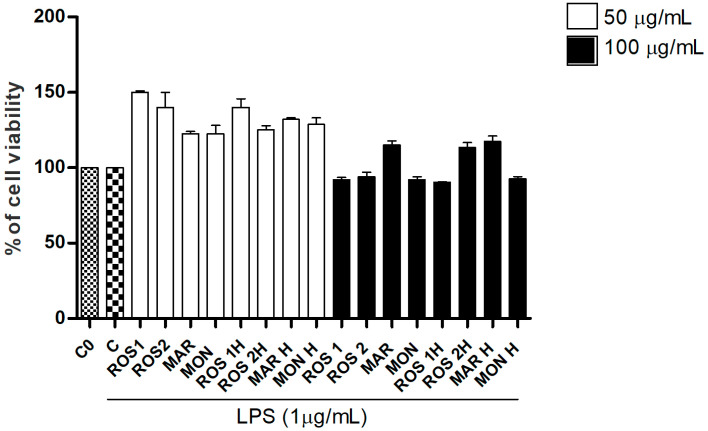
Cell viability of LPS-stimulated RAW 264.7 cells treated with *G. glabra* extracts as evaluated by SRB assay. Abbreviation codes are reported in Table 1. C0: control (untreated cells); C: control (untreated cells) with LPS. Data are expressed as mean ± S.E.M. from four independent experiments (n = 4), each one performed in three technical replicates.

Cells previously tested for mycoplasma negativity (ratio values < 1), were investigated for viability by means of an SRB assay in the presence of *G. glabra* extracts. Two different concentrations, 50 and 100 µg/mL [28], of raw and hydrolyzed extracts were tested in presence of LPS (1 µg/mL). As reported in Figure 1, none of the tested concentrations showed cytotoxic effects on the treated cell line.

**Figure 2:**
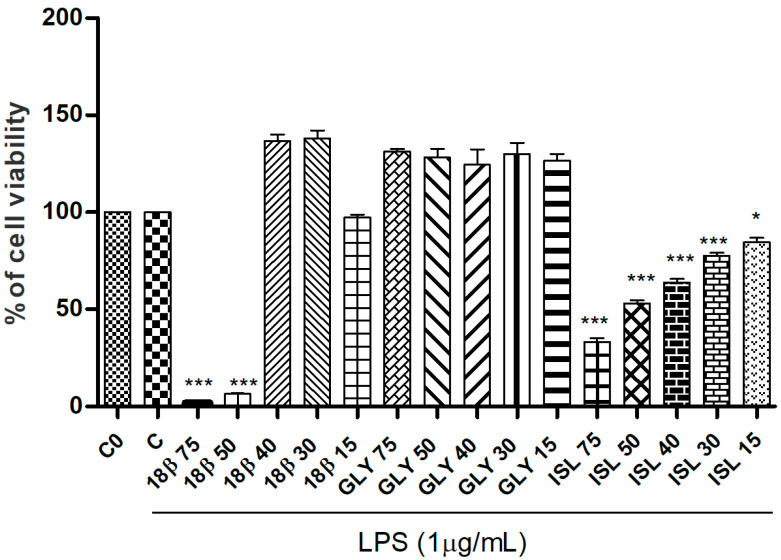
Cell viability of LPS-stimulated RAW 264.7 cells treated with 18β-glycyrrhetinic acid (18β), glycyrrhizin (Gly) and isoliquiritigenin (ISL) (75, 50, 40, 30, 15 µM) evaluated at SRB assay. C0: control (untreated cells); C: control (untreated cells) with LPS. Data are expressed as mean ± S.E.M. from four independent experiments (n = 4), each one performed in three technical replicates. Mean values of samples showing significant difference compared to C were denoted with *** *p* < 0.001, * *p* < 0.05 (Dunnett’s multiple comparison test).

Each investigated licorice standard (18β-glycyrrhetinic acid, glycyrrhizin and isoliquiritigenin) was tested for cell viability at five increasing concentrations (15, 30, 40, 50 and 75 µM) in presence of LPS (1 µg/mL). As shown in Figure 2, glycyrrhizin did not affect cell viability at any tested concentration, while the 18β-glycyrrhetinic acid, at the higher tested doses, 50 and 75 µM, significantly induced a reduction of RAW 264.7 cell viability. As regards isoliquiritigenin, it induced a not negligible cell viability reduction even at the lowest tested dose (15 µM). For this reason, isoliquiritigenin was not selected and carried forward for further biological investigations.

**Figure 3:**
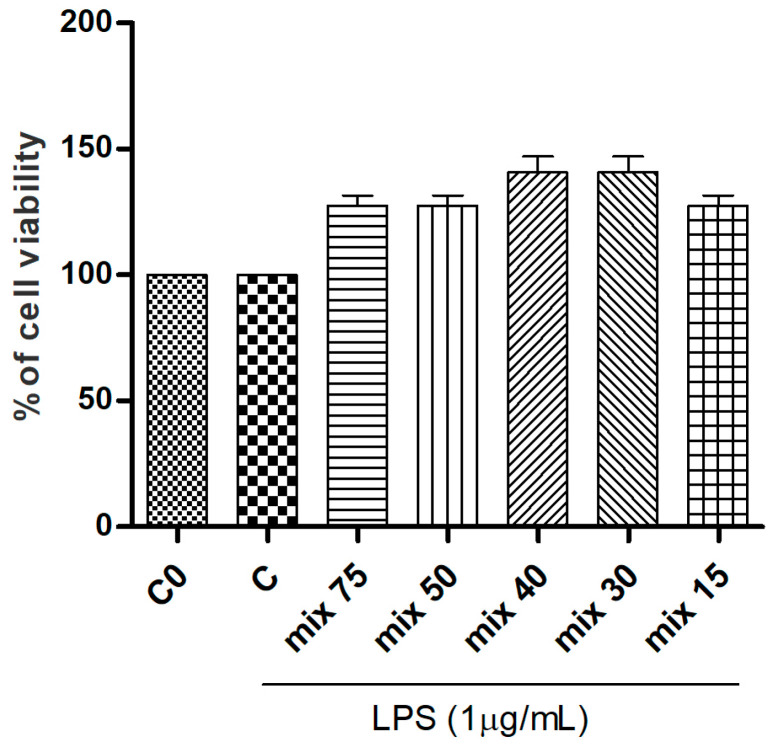
Effects of 18β-glycyrrhetinic acid and glycyrrhizin combinations (Mix) at different concentrations on LPS-stimulated RAW 264.7 cells viability. Combination proportions derive from HPLC detailed ratio (ROS 1 proportion). C0: control (untreated cells); C: control (untreated cells) with LPS. Data are expressed as mean ± S.E.M. from four independent experiments (n = 4), each one performed in three technical replicates.

Starting from the results detailed by HPLC quantification of the standards within the extracts, the main licorice compounds, glycyrrhizin and 18β-glycyrrhetinic acid, respectively, were mixed in a combination mimicking their natural ratio in the raw extracts (previously calculated through HPLC analyses). In order to compare the activity of the pure single standards with these formulated mixtures, the final concentrations corresponded to that of the most abundant compound, glycyrrhizin. The formulated mixtures were tested for cell viability with an SRB assay and, as reported, none of the tested samples induced cytotoixc effects on the RAW 264.7 cell line (Figure 3).

In order to compare pure single standards and the mixture at the same final concentration, the highest non-toxic dose in RAW 264.7 cells was selected for each analyzed sample and carried forward for further investigations. Concerning the extracts, they were analyzed at the highest sub-cytotoxic dose of 100 µg/mL.

### 3.3. Effects of G. glabra L. Extracts and Standards on LPS-Induced Production of Pro-Inflammatory Cytokines (TNF-α, IL-6), the NO Mediator and the Induction of the Anti-Inflammatory Cytokine (IL-10)

**Figure 4:**
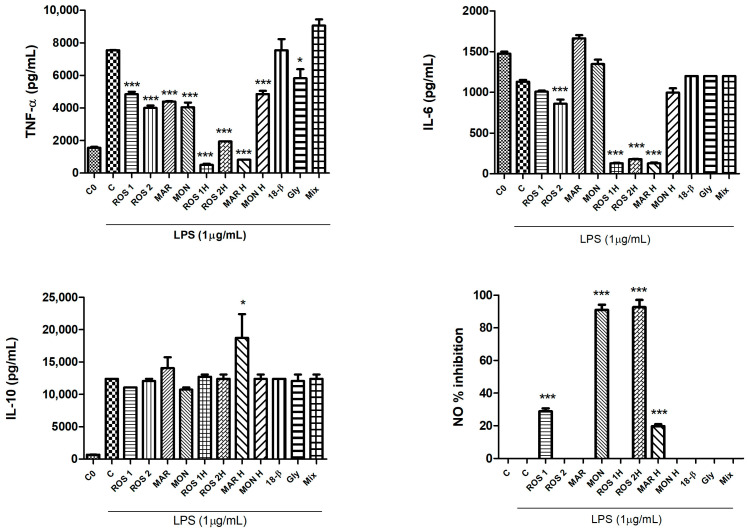
*G. glabra* L. raw and hydrolyzed extracts (100 µg/mL), single standards of 18β-glycyrrhetinic acid (18β) 40 µM and glycyrrhizin (Gly) 40 µM and a mixture of them (Mix) (ROS1 proportion) and their potential inhibitory activity on the release of pro-inflammatory cytokines (TNF-α and IL-6), induction of the anti-inflammatory cytokine IL-10 release and production of nitric oxide (NO) mediator inhibition in LPS-stimulated RAW264.7 cells. C0: control (untreated cells); C: control (untreated cells) with LPS. Significant differences versus control in presence of LPS (C): * *p* < 0.05, *** *p* < 0.001 (Dunnett’s multiple comparison test).

The anti-inflammatory potential of *G. glabra* raw and hydrolyzed extracts, together with that of glycyrrhizin (40 µM), 18β-glycyrrhetinic acid (40 µM) and their mixture (glycyrrhizin and 18β-glycyrrhetinic acid mixed in the proportion detailed by the HPLC ratio for extract) was investigated. The anti-inflammatory potential of both glycyrrhizin and glycyrrhetinic acid was explained through cytokines inhibition and transcription factors (NF-ĸB and STAT3) downregulation [12]. All the hydrolyzed extracts significantly reduced the production of the pro-inflammatory cytokine TNF-α compared to the control in the presence of 1 µg/mL LPS (C). Also, the corresponding raw extracts, even if to a minor extent, significantly inhibited TNF-α release. Among the standards and the mixture, only glycyrrhizin 40 µM significantly inhibited TNF-α release (*p* < 0.05 at Dunnett’s multiple comparison test). Following the same trend, ROS 1H, ROS 2H and MAR H hydrolyzed extracts, as well as ROS 2 raw extract significantly reduced the production and release of IL-6 (*p* < 0.001 at Dunnett’s multiple comparison test). The secretion of the anti-inflammatory cytokine IL-10 was significantly stimulated only by MAR H hydrolyzed extract (*p* < 0.05 at Dunnett’s multiple comparison test). The inhibition of NO oxidized stable products was assessed by ROS 2H and MON *G. glabra* extracts, which had the highest inhibition percentage, followed by ROS 1 and MAR H extracts (inhibition percentage lower than 40%) (Figure 4).

### 3.4. Effects of G. glabra L. Extracts and Standards on LPS-Induced Activation of the JAK/STAT Signaling Pathway

**Figure 5:**
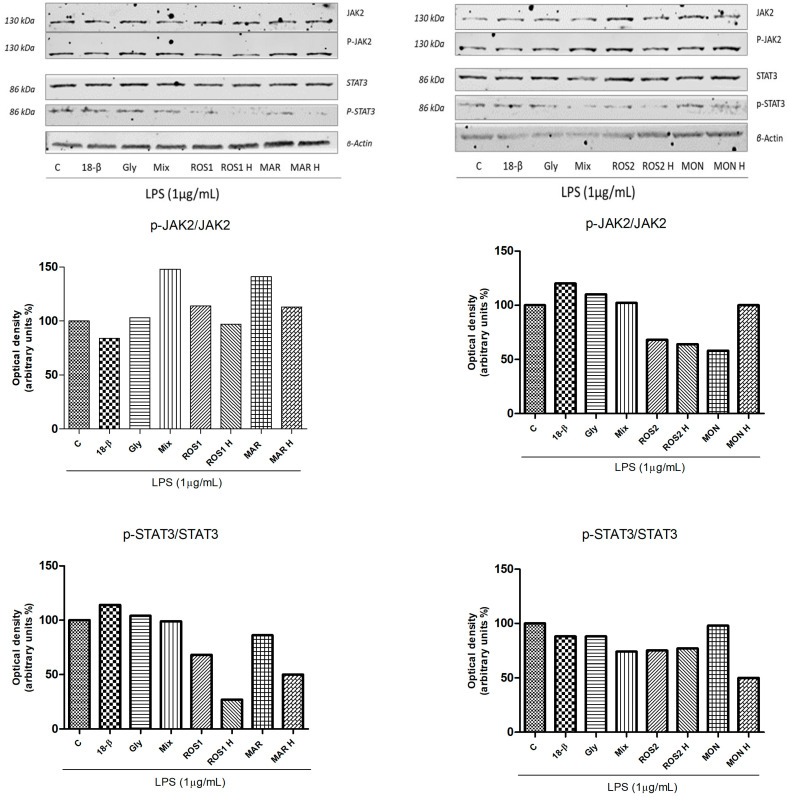
*G. glabra* L. raw and hydrolyzed extracts (100 µg/mL), single standards of 18β-glycyrrhetinic acid 40 µM (18β) and glycyrrhizin 40 µM (Gly) and a mixture of them (Mix) (ROS 1 proportion) and their potential ability to downregulate p-JAK2 and p-STAT3 phosphorylated proteins. Cells were pretreated with samples and stimulated with LPS (1 µg/mL) after 30 min. After 2 h cells were lysed and equal amounts (70 µg) of total cellular extracts were analyzed. β-actin, as a protein not subjected to modulation by treatments, was used as loading control. Histograms refer to the densitometric analyses of the Western blot shown and were obtained by normalizing phosphorylated forms on relative total proteins, previously normalized versus β-actin.

Samples were also tested for their ability to inhibit the phosphorylation of JAK2 and STAT3 proteins through Western blot analyses. 18β-glycyrrhetinic acid (40 µM) and glycyrrhizin (40 µM) as well as ROS 2, ROS 2H and MON inhibited JAK2 phosphorylation, while all raw and hydrolyzed extracts downregulated STAT3 phosphorylated proteins (Figure 5).

As reported by Thiyagarajan and colleagues (2011), *G. glabra* extracts significantly inhibited the production of IL-1β and IL-6 pro-inflammatory cytokines and NO (with an IC_50_ value equal to 24 µg/mL) in J774A.1 murine macrophages stimulated with LPS (0.1 µg/mL). On the contrary, glycyrrhizin did not trigger any anti-inflammatory activity versus LPS-induced mediators [29]. It was already demonstrated that both licorice aqueous extract and one its main components, glycyrrhetinic acid, possess an anti-inflammatory activity comparable with that of diclofenac sodium, one of the main drugs actually used for treatment of inflammatory disease. Moreover, it seems that the formulation of diclofenac and licorice aqueous extract improves the anti-inflammatory potential of the commercial drug on its own. *Glycyrrhiza* ethanolic extracts decreased TNF-α and IL-6 and ameliorated plasma levels of IL-10 cytokine in mice stimulated with LPS [30]. Moreover, the *G. glabra* extracts not containing glycyrrhizin showed interesting anti-inflammatory potential in mammalian cells by inhibiting Prostaglandin E2 (PGE-2), Thromboxane B2 (TXB-2) and Leukotriene B4 (LTB-4) [31]. Another study conducted by Wang and colleagues (2019), showed that glycyrrhizin exerted both anti-inflammatory and analgesic potential by inhibiting the production of Cyclooxygenase-2 (COX-2) and inducible Nitric Oxide Synthase (iNOS) and by decreasing the release of TNF-α and IL-6 cytokines [32]. Bodet and colleagues (2008) showed that the extract from *G. uralensis* inhibited IL-1β, IL-6, IL-8 and TNF-α periodontopathogen triggered by LPS in macrophages [33]. Wang and colleagues (2012) demonstrated that both glycyrrhizin and 18β-glycyrrhetinic acid were effective in the inhibition of NO, PGE_2_ and Reactive Oxygen Species (ROS) and in the downregulation of pro-inflammatory cytokines such as IL-6 and IL-1β in a dose-dependent manner [34]. Zhou and Wink (2019) demonstrated that *G. glabra* extracts inhibited NO induction and NF-ĸB translocation inside the nucleus in LPS-stimulated RAW264.7 macrophages [35]. Furthermore, other studies demonstrated that *G. glabra* ethanolic or methanolic extracts inhibited NO and PGE_2_ production in LPS-activated RAW264.7 cells [36,37]. *G. uralensis* roots from South Korea showed anti-inflammatory potential by suppressing protein expression of IL-1β, IL-6, COX-2 and iNOS in LPS-stimulated RAW264.7 cells and by downregulating NF-ĸB luciferase activity in HT-29 colon cancer cells [38].

These results assumed a central role in prevention. It is already known that prolonged exposure to pro-inflammatory stimuli and mediators, together with the aberrant activation of signal-transduction pathways can promote a condition of mild but chronic inflammation strictly correlated with a plethora of chronic pathologies including metabolic syndrome-related disorders and cancer [39]. All the discussed works highlighted the great anti-inflammatory potential of *G. glabra* extracts and the secondary metabolites they produce, which is in accordance with the data shown in this study.

Each plant species requires specific conditions for its growth and development. On the one hand, genetic factors significantly influence the quality and amounts of secondary metabolites produced; on the other hand, the environmental factors such as cultivation areas, habitat, temperature, light, humidity rate and soil characteristics can affect metabolic pathways, gene expression, and enzymatic activities. A study conducted by Eghlima et al. (2025) compared several different *G. glabra* samples collected in various Uzbekistan regions; data showed that the glycyrrhizic acid content may vary from 3.3% to 6.1% of dry weight, while glabridin amounts range from 0.08% to 0.35% of dry weight [26]. A study by Montoro and colleagues (2011) investigated the glycyrrhizic acid amount extracted from *G. glabra* root samples from Italy, China, Turkey and Iran: The data showed significant differences in concentrations, namely 51 mg/g, 53 mg/g, 33 mg/g and 32 mg/g of dry weight, respectively [40]. Glycyrrhizin content detected in the Zagros region (Iran) showed amount ranging from 0.03% to 0.23%. A study conducted by Semenescu et al. (2024) investigated the phytochemical profile and the biological activities of *G. glabra* samples collected in Romania [41]. Among the secondary metabolites identified, glycyrrhizin was detected during HPLC analyses at 13.927 mg/g.

## 4. Conclusions

Over the past years, *G. glabra* extracts, as well as their secondary metabolites, were largely studied for their interesting biological properties. In this paper, the anti-inflammatory potential activities of both the extracts and their main standards (glycyrrhizin, 18β-glycyrrhetinic acid and isoliquiritigenin) were investigated and compared with each other. Here, the best efficacy in inhibiting the production of pro-inflammatory cytokines (TNF-α, IL-6) and NO mediator and the best downregulation of the JAK/STAT signaling pathway was attributable to *G. glabra* extracts if compared to the standards. Among the extracts, the best activity was exerted by the hydrolyzed extracts, if compared to the raw ones; this process mainly involves glycyrrhizin, a triterpene saponin considered to be the most abundant compound in licorice that hydrolyzes in 18β-glycyrrhetinic acid. In particular, MAR H, by affecting all the investigated pro-inflammatory stimuli (TNF-α, IL-6), NO mediator and phosphorylation of JAK2 and STAT3 proteins, and being the only sample inducing a significant release of the IL-10 anti-inflammatory cytokine, can be considered the most effective extract. In conclusion, in this article, the potential anti-inflammatory activity of *G. glabra* extracts and of their main standards were investigated, suggesting they could represent potential valuable candidates for the treatment of inflammatory diseases acting through the inhibition of the JAK/STAT signaling pathway.

## Figures and Tables

**Table 1:** *G. glabra* geographical area origin, starting material and abbreviation codes.

*G. glabra* Sample Origin	Material	Raw/Hydrolyzed Extracts Codes	Coordinates
Montalto Uffugo (CS), Italy	Collected	MON/MON H	39°24′ N; 16°09′ E
Rossano (CS), Italy	Commercially available	ROS1/ROS1 H	-
Rossano (CS), Italy	Commercially available	ROS2/ROS2 H	-
Morocco	Commercially available	MAR/MAR H	-

## Data Availability

The data presented in this study are available in the article.

## Supplementary Materials

The following supporting information can be downloaded at https://www.mdpi.com/article/10.3390/foods14213746/s1, Table S1: 18β-glycyrrhetinic acid, Isoliquiritigenin and Glycyrrhizin HPLC determination and quantification in *G. glabra* samples collected in different geographical areas.

## Abbreviations

The following abbreviations are used in this manuscript:

EMA	European Medicine Agency
NSAIDs	Non-steroidal anti-inflammatory drugs
TNF-α	Tumor Necrosis Factor-α
IL-6	Interleukin-6
NO	Nitric Oxide
IL-10	Interleukin-10
LPS	Lipopolysaccharide
JAK2	Janus Kinase
STAT-3	Signal Transducer and Activator of Transcription 3
11β-HSD2	11 beta-hydroxysteroid dehydrogenase
FDA	Food and Drug Administration
GRAS	Generally Recognized As Safe
ADI	Admitted Daily Intake
HPLC	High Performance Liquid Chromatography
DMEM	Dulbecco’s Modified Eagle’s Medium
FBS	Fetal Bovin Serum
PBS	Phosphate Buffered Saline
BSA	Bovine Serum Albumin
TCA	Trichloroacetic Acid
SRB	Sulphorhodamine B
ISL	Isoliquiritigenin
Gly	Glycyrrhizin
18-β	18-β glycyrrhetinic acid
PGE-2	Prostaglandin E2
TXB-2	Thromboxane B2
LTB-4	Leukotriene B4
COX-2	Cyclooxygenase-2
iNOS	Inducible Nitrix Oxide Synthase
ROS	Reactive Oxygen Species
