# Supplementary material for: Glycyrrhiza glabra L. Extracts Prevent LPS-Induced Inflammation in RAW264.7 Cells by Targeting Pro-Inflammatory Cytokines, Mediators and the JAK/STAT Signaling Pathway
Source: Foods. 2025 Oct 31;14(21):3746. doi: 10.3390/foods14213746 (PMC12610661; doi:10.3390/foods14213746)
Supplement: Supplementary file 1 [file foods-14-03746-s001.zip › foods-3916522-supplementary.pdf]

Table S1. *18*β-glycyrrhetinic acid, Isoliquiritigenin and Glycyrrhizin HPLC determination and quantification in *G. glabra* samples collected in different geographical areas

**Samples**	**18β-glycyrrhetinic acid (μg/1mg of extract)**	**Isoliquiritigenin (μg/1mg of extract)**	**Glycyrrhizin (μg/1mg of extract)**
ROS1	0.57 ± 0.303[e]	1.08 ± 0.065[e]	62.09 ± 1.153[c]
ROS2	0.37 ± 0.007[e]	1.40 ± 0.072[e]	63.25 ± 1.725[c]
MAR	0.21 ± 0.029[e]	0.80 ± 0.019[e]	70.83 ± 1.985[b]
MON	n.d.*	0.25 ± 0.022[e]	78.04 ± 0.760[a]
ROS 1H	33 ± 0.916[c]	29 ± 1.581[c]	n.d.
ROS 2H	30 ± 0.320[d]	38 ± 0.036[a]	n.d.
MAR H	38 ± 0.528[b]	33 ± 0.138[b]	n.d.
MON H	40 ± 0.834[a]	19 ± 0.887[d]	n.d.

Results were expressed as mean ± S.D. (n=3). Data are expressed as μg/1mg of extracts. Different letters along column indicate statistical differences at $p < 0.05$ (Bonferroni post-hoc test). *n.d.: not detected. Abbreviation codes are reported in Table 1.
